# *trans*-Diaqua­bis­[4-carboxy-5-carboxyl­ato-2-(pyridin-1-ium-4-yl)-1*H*-imidazol-1-ido-κ^2^ *N* ^1^,*O* ^5^]cobalt(II)

**DOI:** 10.1107/S1600536811032545

**Published:** 2011-08-17

**Authors:** Lin Sun, Yu Hua Huang, Ting Ting Chen, Hong Deng

**Affiliations:** a School of Chemistry and Environment, South China Normal University, Guangzhou 510006, People’s Republic of China

## Abstract

In the title compound, [Co(C_10_H_6_N_3_O_4_)_2_(H_2_O)_2_], the Co^II^ ion is coordinated by two O atoms of two water mol­ecules, two imidazole nitro­gen atoms and two carboxyl­ate O atoms of the two *trans*-standing chelate ligands, displaying a distorted octa­hedral coordination geometry. A three-dimensional supra­molecular framework is generated through N—H⋯O, O—H⋯N and O—H⋯O hydrogen-bonding inter­actions.

## Related literature

For the chemistry of *N*-heterocyclic carb­oxy­lic acids, see: Peng *et al.* (2010 ▶); Liu *et al.* (2005 ▶). For the applications of 2-(pyridine-4-yl)-1*H*-4,5-imidazole­dicarb­oxy­lic acid, see: Li, Liu *et al.* (2009 ▶); Sun *et al.* (2006 ▶); Li, Wu *et al.* (2009 ▶); Chen *et al.* (2010 ▶).
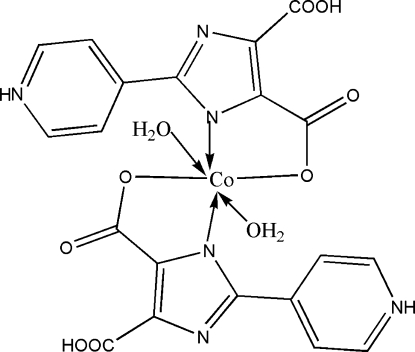


## Experimental

### 

#### Crystal data

[Co(C_10_H_6_N_3_O_4_)_2_(H_2_O)_2_]
*M* *_r_* = 559.32
Monoclinic, 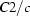

*a* = 7.4146 (17) Å
*b* = 20.190 (5) Å
*c* = 13.361 (3) Å
β = 97.383 (3)°
*V* = 1983.6 (8) Å^3^
*Z* = 4
Mo *K*α radiation
μ = 0.95 mm^−1^
*T* = 296 K
0.27 × 0.26 × 0.24 mm

#### Data collection

Bruker AXS SMART APEX CCD diffractometer
Absorption correction: multi-scan (*SADABS*; Sheldrick, 1996 ▶) *T*_min_ = 0.775, *T*_max_ = 0.797
4997 measured reflections
1775 independent reflections
1480 reflections with *I* > 2σ(*I*)
*R*_int_ = 0.032

#### Refinement

*R*[*F* ^2^ > 2σ(*F* ^2^)] = 0.035
*wR*(*F* ^2^) = 0.088
*S* = 1.01
1775 reflections
181 parameters
3 restraints
H atoms treated by a mixture of independent and constrained refinement
Δρ_max_ = 0.27 e Å^−3^
Δρ_min_ = −0.43 e Å^−3^

### 

Data collection: *APEX2* (Bruker, 2004 ▶); cell refinement: *SAINT* (Bruker, 2004 ▶); data reduction: *SAINT*; program(s) used to solve structure: *SHELXS97* (Sheldrick, 2008 ▶); program(s) used to refine structure: *SHELXL97* (Sheldrick, 2008 ▶); molecular graphics: *SHELXTL* (Sheldrick, 2008 ▶); software used to prepare material for publication: *SHELXTL*.

## Supplementary Material

Crystal structure: contains datablock(s) I, global. DOI: 10.1107/S1600536811032545/bt5574sup1.cif

Structure factors: contains datablock(s) I. DOI: 10.1107/S1600536811032545/bt5574Isup2.hkl

Additional supplementary materials:  crystallographic information; 3D view; checkCIF report

## Figures and Tables

**Table 1:** Hydrogen-bond geometry (Å, °)

*D*—H⋯*A*	*D*—H	H⋯*A*	*D*⋯*A*	*D*—H⋯*A*
O1*W*—H1*WA*⋯N2^i^	0.84 (2)	2.05 (2)	2.889 (3)	176 (4)
O1*W*—H1*WB*⋯O4^ii^	0.81 (2)	2.27 (2)	3.046 (3)	161 (4)
N3—H3⋯O4^iii^	0.91 (3)	1.86 (3)	2.754 (3)	170 (3)
O3—H3*A*⋯O2	1.20 (4)	1.25 (4)	2.451 (3)	172 (3)

Symmetry codes: (i) 
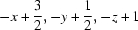
; (ii) 
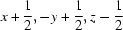
; (iii) 
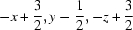
.

**Fig. 1.**
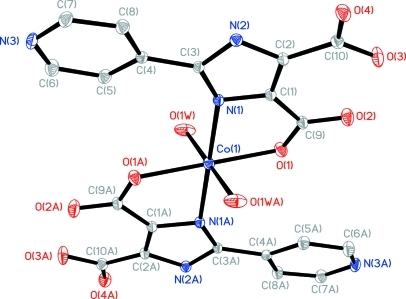
The structure of (I), showing the atomic numbering scheme. Non-H atoms are shown as 50% probability displacement ellipsoids.

**Fig. 2.**
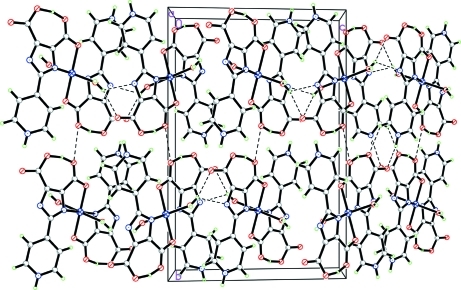
A packing view of (I) along the a axis, showing the hydrogen bonds.

## supplementary crystallographic information

### Comment

Mixed N– and O-donor organic ligands, such as 1*H*-benzimidazole-5-carboxylic acid, has been investigated and proved to be a good choice to costruct novel coordination polymers (Peng *et al.*, 2010; Liu *et al.*, 2005). Another hybrid molecule 2-(pyridine-4-yl)-1*H*-4,5-imidazoledicarboxylic acid exhibits diverse coordination modes for its abundant potential donor atoms (Li, Liu *et al.*, 2009; Sun *et al.*, 2006; Li, Wu *et al.*, 2009; Chen *et al.*, 2010). In this work, we employed 2-(pyridine-4-yl)-1*H*-4,5-imidazoledicarboxylic acid as the building blocks to synthesize the title compound under hydrothermal condition. The centre metal ion Co^ii^ lies on an inversion centre and is coordinated by two imidazole nitrogen atoms, two O atoms of two water molecules and two carboxylate O atoms of the two *trans*-standing chelate ligands, displaying a distorted octahedral coordination geometry. The bond distances of Co—O_w_ and Co—O_carboxylate_ are 2.092 (3)Å and 2.066 (3) Å, respectively. And the Co—N bond length is 2.168 (3)Å (Table 1). The dihedral angle between the pyridyl ring and the imidazole ring is about 13.98 (2)° (Table 1, Figure 1). Since there are many hydrogen bonding interactions, a three-dimensional supramolecular architecture is eventually formed *via* N—H···O, O—H···N and O—H···O hydrogen-bonding interactions (Table 2, Figure 2).

### Experimental

A mixture of CoSO_4_(0.4 mmol, 0.062 g) and 2-(pyridine-4-yl)-1*H*-4,5-imidazoledicarboxylic acid (0.5 mmol, 0.115 g) together with water (10 ml) was sealed in a 23 ml Teflon-lined stainless steel reactor and heated at 160°C under autogenous pressure for 96 h. Then the mixture was cooled down to room temperature at a rate of 5°C/h, and brown block crystals were obtained in a yield of 39% based on Co.

### Refinement

H atoms bonded to C  atoms   were  ideally positioned with C—H=0.93 Å, and displacement parameters set to 1.2 times of their carrier atoms. All H atoms attached to O and N atoms were located in difference density Fourier maps and H atoms of water were refined with the following distance restraints:  O—H = 0.82 (2) Å and H—H = 1.35 (2) Å with *U*_iso_(H) = 1.5 *U*_eq_(O) and *U*_iso_(H) = 1.2*U*_eq_(N) respectively.

### Crystal data

**Table:**

[Co(C_10_H_6_N_3_O_4_)_2_(H_2_O)_2_]	*F*(000) = 1140
*M**_r_* = 559.32	*D*_x_ = 1.873 Mg m^−^^3^
Monoclinic, *C*2/*c*	Mo *K*α radiation, λ = 0.71073 Å
Hall symbol: -C 2yc	Cell parameters from 1775 reflections
*a* = 7.4146 (17) Å	θ = 2.5–25.5°
*b* = 20.190 (5) Å	µ = 0.95 mm^−^^1^
*c* = 13.361 (3) Å	*T* = 296 K
β = 97.383 (3)°	Block, brown
*V* = 1983.6 (8) Å^3^	0.27 × 0.26 × 0.24 mm
*Z* = 4	

### Data collection

**Table:**

Bruker AXS SMART APEX CCD diffractometer	1775 independent reflections
Radiation source: fine-focus sealed tube	1480 reflections with *I* > 2σ(*I*)
graphite	*R*_int_ = 0.032
ω scans	θ_max_ = 25.2°, θ_min_ = 2.0°
Absorption correction: multi-scan (*SADABS*; Sheldrick, 1996)	*h* = −8→8
*T*_min_ = 0.775, *T*_max_ = 0.797	*k* = −17→24
4997 measured reflections	*l* = −15→15

### Refinement

**Table:**

Refinement on *F*^2^	Primary atom site location: structure-invariant direct methods
Least-squares matrix: full	Secondary atom site location: difference Fourier map
*R*[*F*^2^ > 2σ(*F*^2^)] = 0.035	Hydrogen site location: inferred from neighbouring sites
*wR*(*F*^2^) = 0.088	H atoms treated by a mixture of independent and constrained refinement
*S* = 1.01	*w* = 1/[σ^2^(*F*_o_^2^) + (0.034*P*)^2^ + 5.2791*P*] where *P* = (*F*_o_^2^ + 2*F*_c_^2^)/3
1775 reflections	(Δ/σ)_max_ = 0.021
181 parameters	Δρ_max_ = 0.27 e Å^−^^3^
3 restraints	Δρ_min_ = −0.43 e Å^−^^3^

### Special details

**Table:**

Geometry. All e.s.d.'s (except the e.s.d. in the dihedral angle between two l.s. planes) are estimated using the full covariance matrix. The cell e.s.d.'s are taken into account individually in the estimation of e.s.d.'s in distances, angles and torsion angles; correlations between e.s.d.'s in cell parameters are only used when they are defined by crystal symmetry. An approximate (isotropic) treatment of cell e.s.d.'s is used for estimating e.s.d.'s involving l.s. planes.
Refinement. Refinement of *F*^2^ against ALL reflections. The weighted *R*-factor *wR* and goodness of fit *S* are based on *F*^2^, conventional *R*-factors *R* are based on *F*, with *F* set to zero for negative *F*^2^. The threshold expression of *F*^2^ > σ(*F*^2^) is used only for calculating *R*-factors(gt) *etc*. and is not relevant to the choice of reflections for refinement. *R*-factors based on *F*^2^ are statistically about twice as large as those based on *F*, and *R*- factors based on ALL data will be even larger.

### Fractional atomic coordinates and isotropic or equivalent isotropic displacement parameters (Å^2^)

**Table:**

	*x*	*y*	*z*	*U*_iso_*/*U*_eq_	
C1	0.9792 (4)	0.32925 (12)	0.57368 (19)	0.0187 (6)	
C2	0.8527 (4)	0.34198 (12)	0.63981 (19)	0.0193 (6)	
C3	0.8791 (3)	0.23737 (12)	0.62348 (19)	0.0178 (5)	
C4	0.8456 (3)	0.16716 (13)	0.63859 (19)	0.0193 (6)	
C5	0.9082 (4)	0.11718 (14)	0.5791 (2)	0.0257 (6)	
H5	0.9695	0.1279	0.5248	0.031*	
C6	0.8781 (4)	0.05252 (15)	0.6016 (2)	0.0332 (7)	
H6	0.9176	0.0193	0.5613	0.040*	
C7	0.7298 (4)	0.08204 (14)	0.7376 (2)	0.0282 (7)	
H7	0.6702	0.0695	0.7918	0.034*	
C8	0.7511 (4)	0.14764 (13)	0.7177 (2)	0.0254 (6)	
H8	0.7026	0.1795	0.7569	0.031*	
C9	1.0837 (4)	0.37354 (13)	0.5147 (2)	0.0234 (6)	
C10	0.7965 (4)	0.40581 (13)	0.6804 (2)	0.0240 (6)	
Co1	1.2500	0.2500	0.5000	0.02030 (17)	
N1	0.9989 (3)	0.26264 (10)	0.56502 (16)	0.0176 (5)	
N2	0.7881 (3)	0.28357 (10)	0.67008 (16)	0.0203 (5)	
N3	0.7934 (3)	0.03578 (12)	0.68026 (19)	0.0290 (6)	
H3	0.779 (4)	−0.0073 (16)	0.697 (2)	0.035*	
O1	1.1926 (3)	0.34767 (9)	0.46237 (15)	0.0295 (5)	
O2	1.0579 (3)	0.43564 (9)	0.51720 (17)	0.0358 (5)	
O3	0.8438 (3)	0.45907 (9)	0.63801 (17)	0.0376 (6)	
O4	0.7109 (3)	0.40657 (10)	0.75400 (16)	0.0330 (5)	
O1W	1.1046 (3)	0.21633 (13)	0.36503 (16)	0.0390 (6)	
H1WA	0.990 (2)	0.2183 (19)	0.356 (3)	0.058*	
H1WB	1.139 (4)	0.1901 (16)	0.326 (2)	0.058*	
H3A	0.940 (5)	0.4481 (18)	0.574 (3)	0.058*	

### Atomic displacement parameters (Å^2^)

**Table:**

	*U*^11^	*U*^22^	*U*^33^	*U*^12^	*U*^13^	*U*^23^
C1	0.0225 (14)	0.0126 (13)	0.0219 (14)	0.0016 (11)	0.0061 (11)	0.0013 (10)
C2	0.0237 (14)	0.0142 (13)	0.0208 (14)	0.0007 (10)	0.0061 (11)	0.0008 (10)
C3	0.0203 (13)	0.0141 (13)	0.0197 (13)	−0.0001 (10)	0.0048 (10)	0.0018 (10)
C4	0.0196 (14)	0.0175 (14)	0.0207 (14)	−0.0002 (11)	0.0019 (10)	0.0022 (11)
C5	0.0315 (15)	0.0212 (15)	0.0262 (15)	−0.0021 (12)	0.0106 (12)	0.0008 (11)
C6	0.0450 (18)	0.0211 (15)	0.0357 (18)	−0.0007 (13)	0.0136 (14)	−0.0048 (13)
C7	0.0337 (17)	0.0229 (15)	0.0304 (16)	−0.0024 (12)	0.0127 (13)	0.0049 (12)
C8	0.0307 (15)	0.0193 (14)	0.0285 (15)	0.0002 (12)	0.0124 (12)	0.0002 (12)
C9	0.0269 (15)	0.0187 (14)	0.0259 (15)	0.0008 (11)	0.0081 (12)	0.0029 (11)
C10	0.0260 (15)	0.0192 (14)	0.0280 (16)	0.0015 (12)	0.0076 (12)	−0.0032 (11)
Co1	0.0246 (3)	0.0161 (3)	0.0218 (3)	0.0021 (2)	0.0090 (2)	0.0006 (2)
N1	0.0215 (11)	0.0137 (11)	0.0187 (11)	0.0008 (9)	0.0065 (9)	0.0009 (8)
N2	0.0229 (12)	0.0159 (11)	0.0235 (12)	0.0006 (9)	0.0079 (9)	0.0007 (9)
N3	0.0361 (14)	0.0144 (12)	0.0375 (15)	−0.0041 (11)	0.0083 (11)	0.0038 (11)
O1	0.0361 (12)	0.0200 (10)	0.0365 (12)	0.0046 (9)	0.0201 (9)	0.0075 (9)
O2	0.0500 (14)	0.0132 (10)	0.0498 (14)	0.0029 (9)	0.0275 (11)	0.0058 (9)
O3	0.0600 (15)	0.0148 (10)	0.0439 (13)	0.0026 (10)	0.0285 (11)	−0.0002 (9)
O4	0.0425 (13)	0.0223 (11)	0.0383 (12)	0.0023 (9)	0.0211 (10)	−0.0056 (9)
O1W	0.0258 (11)	0.0621 (17)	0.0296 (12)	0.0044 (11)	0.0056 (10)	−0.0179 (11)

### Geometric parameters (Å, °)

**Table:**

C1—N1	1.359 (3)	C8—H8	0.9300
C1—C2	1.393 (4)	C9—O1	1.248 (3)
C1—C9	1.475 (4)	C9—O2	1.269 (3)
C2—N2	1.354 (3)	C10—O4	1.237 (3)
C2—C10	1.479 (4)	C10—O3	1.285 (3)
C3—N2	1.350 (3)	Co1—O1^i^	2.0659 (19)
C3—N1	1.355 (3)	Co1—O1	2.0659 (19)
C3—C4	1.458 (3)	Co1—O1W^i^	2.092 (2)
C4—C8	1.398 (4)	Co1—O1W	2.092 (2)
C4—C5	1.400 (4)	Co1—N1^i^	2.169 (2)
C5—C6	1.365 (4)	Co1—N1	2.169 (2)
C5—H5	0.9300	N3—H3	0.91 (3)
C6—N3	1.335 (4)	O2—H3A	1.25 (4)
C6—H6	0.9300	O3—H3A	1.20 (4)
C7—N3	1.332 (4)	O1W—H1WA	0.841 (18)
C7—C8	1.364 (4)	O1W—H1WB	0.810 (18)
C7—H7	0.9300		
			
N1—C1—C2	109.0 (2)	O3—C10—C2	117.5 (2)
N1—C1—C9	119.0 (2)	O1^i^—Co1—O1	180.00 (11)
C2—C1—C9	132.0 (2)	O1^i^—Co1—O1W^i^	91.89 (9)
N2—C2—C1	108.8 (2)	O1—Co1—O1W^i^	88.11 (9)
N2—C2—C10	121.5 (2)	O1^i^—Co1—O1W	88.11 (9)
C1—C2—C10	129.6 (2)	O1—Co1—O1W	91.89 (9)
N2—C3—N1	114.1 (2)	O1W^i^—Co1—O1W	180.0
N2—C3—C4	120.3 (2)	O1^i^—Co1—N1^i^	79.89 (7)
N1—C3—C4	125.6 (2)	O1—Co1—N1^i^	100.11 (7)
C8—C4—C5	117.5 (2)	O1W^i^—Co1—N1^i^	90.55 (8)
C8—C4—C3	119.3 (2)	O1W—Co1—N1^i^	89.45 (8)
C5—C4—C3	123.2 (2)	O1^i^—Co1—N1	100.11 (7)
C6—C5—C4	119.2 (3)	O1—Co1—N1	79.89 (7)
C6—C5—H5	120.4	O1W^i^—Co1—N1	89.45 (8)
C4—C5—H5	120.4	O1W—Co1—N1	90.55 (8)
N3—C6—C5	121.5 (3)	N1^i^—Co1—N1	180.0
N3—C6—H6	119.2	C3—N1—C1	103.8 (2)
C5—C6—H6	119.2	C3—N1—Co1	147.82 (17)
N3—C7—C8	120.7 (3)	C1—N1—Co1	104.92 (16)
N3—C7—H7	119.7	C3—N2—C2	104.3 (2)
C8—C7—H7	119.7	C7—N3—C6	120.8 (3)
C7—C8—C4	120.2 (3)	C7—N3—H3	118 (2)
C7—C8—H8	119.9	C6—N3—H3	121 (2)
C4—C8—H8	119.9	C9—O1—Co1	113.00 (17)
O1—C9—O2	122.6 (2)	C9—O2—H3A	109.5 (17)
O1—C9—C1	117.9 (2)	C10—O3—H3A	112.3 (17)
O2—C9—C1	119.6 (2)	Co1—O1W—H1WA	121 (2)
O4—C10—O3	122.5 (2)	Co1—O1W—H1WB	127 (2)
O4—C10—C2	120.0 (2)	H1WA—O1W—H1WB	109 (3)

Symmetry codes: (i) −*x*+5/2, −*y*+1/2, −*z*+1.

### Hydrogen-bond geometry (Å, °)

**Table:**

*D*—H···*A*	*D*—H	H···*A*	*D*···*A*	*D*—H···*A*
O1W—H1WA···N2^ii^	0.84 (2)	2.05 (2)	2.889 (3)	176 (4)
O1W—H1WB···O4^iii^	0.81 (2)	2.27 (2)	3.046 (3)	161 (4)
N3—H3···O4^iv^	0.91 (3)	1.86 (3)	2.754 (3)	170 (3)
O3—H3A···O2	1.20 (4)	1.25 (4)	2.451 (3)	172 (3)

Symmetry codes: (ii) −*x*+3/2, −*y*+1/2, −*z*+1; (iii) *x*+1/2, −*y*+1/2, *z*−1/2; (iv) −*x*+3/2, *y*−1/2, −*z*+3/2.
